# Ensuring accurate testing for human immunodeficiency virus in Myanmar

**DOI:** 10.2471/BLT.14.138909

**Published:** 2014-10-24

**Authors:** Latt Latt Kyaw, Ikuma Nozaki, Koji Wada, Khin Yi Oo, Htay Htay Tin, Namiko Yoshihara

**Affiliations:** a National Health Laboratory, Ministry of Health, Yangon, Myanmar.; b Bureau of International Medical Cooperation, National Center for Global Health and Medicine, 1-21-1 Toyama, Shinjuku-ku, Tokyo, Japan.; c JICA Major Infectious Disease Control Project II, Yangon, Myanmar.

## Abstract

**Problem:**

Until 2005, the quality of rapid diagnostic human immunodeficiency virus (HIV) testing was not monitored and no regular technical support was provided to hospital laboratories in Myanmar.

**Approach:**

The national reference laboratory introduced a national external quality assessment scheme. The scheme involved (i) training laboratory technicians in HIV testing and in the requirements of the quality assessment system; (ii) implementing a biannual proficiency panel testing programme; (iii) on-site assessments of poorly-performing laboratories to improve testing procedures; and (iv) development of national guidelines.

**Local setting:**

In 2011, a total of 422 public hospitals in Myanmar had laboratories providing HIV tests. In addition, private laboratories supported by nongovernmental organizations (NGOs) conducted HIV testing.

**Relevant changes:**

The scheme was started in 65 public laboratories in 2005. In 2012, it had expanded nationwide to 347 laboratories, including 33 NGO laboratories. During the expansion of the scheme, laboratory response rates were greater than 90% and the proportion of laboratories reporting at least one aberrant result improved from 9.2% (6/65) in 2005 to 5.4% (17/316) in 2012.

**Lessons learnt:**

National testing guidelines and a reference laboratory are needed to successfully implement quality assurance of HIV testing services. On-site assessments are crucial for all participating laboratories and the only source for insight on the causes of aberrant results; lessons that the reference laboratory can share nationally. Proficiency testing helps laboratory technicians to maintain HIV testing skills by ensuring that they regularly encountered HIV-positive samples.

## Résumé

**Problème:**

Jusqu'à 2005, la qualité du dépistage du virus de l'immunodéficience humaine (VIH) à diagnostic rapide n'était pas surveillée, et aucune assistance technique régulière n'était fournie aux laboratoires hospitaliers du Myanmar.

**Approche:**

Le laboratoire national de référence a mis en place un système national d'évaluation de la qualité externe. Le système impliquait (i) la formation des techniciens de laboratoire au dépistage du VIH et aux exigences du système d'évaluation de la qualité; (ii) la mise en place d'un programme de contrôle des compétences deux fois par an; (iii) l'évaluation sur site des laboratoires à performance médiocre pour améliorer les procédures de dépistage; et (iv) l'élaboration de directives nationales.

**Environnement local:**

En 2011, un total de 422 hôpitaux publics au Myanmar disposaient de laboratoires réalisant des dépistages du VIH. En outre, des laboratoires privés soutenus par des organisations non gouvernementales (ONG) ont également effectué des dépistages du VIH.

**Changements significatifs:**

Le système a été lancé dans 65 laboratoires publics en 2005. En 2012, il a été étendu à l'échelle du pays dans 347 laboratoires, y compris 33 laboratoires gérés par des ONG. Pendant le développement du système, les taux de réponse des laboratoires étaient supérieurs à 90%, et le pourcentage de laboratoire ayant signalé au moins un résultat aberrant s'est amélioré, passant de 9,2% (6/65) en 2005 à 5,4% (17/316) en 2012.

**Leçons tirées:**

Des directives nationales en matière de dépistage et un laboratoire de référence sont nécessaires pour réussir la mise en œuvre de l'assurance qualité des services de dépistage du VIH. Les évaluations sur site sont essentielles pour tous les laboratoires participants et la seule source pour connaître les causes des résultats aberrants. Ce sont des leçons que le laboratoire de référence peut diffuser à l'échelle nationale. Les contrôles de compétence peuvent aider les techniciens de laboratoire à maintenir à niveau leurs compétences en matière de dépistage du VIH en s'assurant qu'ils rencontrent régulièrement des échantillons de VIH séropositifs.

## Resumen

**Situación:**

Hasta 2005, no se había controlado la calidad de las pruebas de diagnóstico rápido del virus de inmunodeficiencia humana (VIH) ni se había proporcionado asistencia técnica constante a los laboratorios de los hospitales en Myanmar.

**Enfoque:**

El laboratorio nacional de referencia introdujo un sistema nacional de evaluación externa de la calidad. El plan incluía (i) la capacitación de técnicos de laboratorio en las pruebas del VIH y en los requisitos del sistema de evaluación de la calidad; (ii) la aplicación de un programa bianual de un cuadro de análisis de la competencia; (iii) evaluaciones *in situ* de los laboratorios con un rendimiento bajo para mejorar los procedimientos de prueba; y (iv) el desarrollo de directrices nacionales.

**Marco regional:**

En 2011, un total de 422 hospitales públicos en Myanmar contaban con laboratorios que ofrecían pruebas del VIH. Además, laboratorios privados apoyados por organizaciones no gubernamentales (ONG) también realizaban pruebas del VIH.

**Cambios importantes:**

El plan se inició en 65 laboratorios públicos en 2005. En 2012, se amplió a nivel nacional a 347 laboratorios, de los cuales, 33 eran laboratorios de ONG. Durante la ampliación del plan, las tasas de respuesta de laboratorio fueron superiores al 90% y la proporción de laboratorios que notificaban al menos un resultado aberrante mejoró del 9,2% (6/65) en 2005 al 5,4% (17/316) en 2012.

**Lecciones aprendidas:**

Se necesitan directrices nacionales para la realización de pruebas y un laboratorio de referencia para aplicar con éxito el control de calidad de los servicios de pruebas del VIH. Las evaluaciones* in situ* son fundamentales para todos los laboratorios participantes y la única fuente para comprender las causas de los resultados anómalos. El laboratorio de referencia puede compartir estas lecciones a nivel nacional. La evaluación de la competencia ayuda a los técnicos de laboratorio a mantener las aptitudes para la realización de las pruebas del VIH, ya que les garantiza encontrar muestras seropositivas.

## ملخص

**المشكلة:**

حتى عام 2005، لم يكن هناك رصد لجودة اختبارات فيروس العوز المناعي البشري التشخيصية السريعة ولم يتم تقديم دعم تقني إلى مختبرات المستشفيات في ميانمار.

**الأسلوب:**

عرض المختبر المرجعي الوطني مخططاً لتقييم الجودة الخارجية على الصعيد الوطني. وتضمن المخطط (1) تدريب فنيي المختبرات على اختبارات فيروس العوز المناعي البشري وعلى متطلبات نظام تقييم الجودة؛ (2) تنفيذ برنامج نصف سنوي لاختبار مستلزمات الكفاءة؛ (3) تقييمات تنفذ في مواقع المختبرات ذات الأداء الضعيف بغية تحسين إجراءات الاختبار؛ (4) وضع دلائل إرشادية وطنية.

**المواقع المحلية:**

في عام 2011، كان ما مجموعه 422 مستشفى عمومياً في ميانمار تحتوي على مختبرات تقدم اختبارات فيروس العوز المناعي البشري. بالإضافة إلى ذلك، أجرت مختبرات خاصة تدعمها منظمات غير حكومية اختبارات فيروس العوز المناعي البشري.

**التغيّرات ذات الصلة:**

تم بدء المخطط في 65 مختبراً عمومياً في عام 2005. وفي عام 2012، شهد المخطط توسعاً على الصعيد الوطني ليشمل 347 مختبراً، بما في ذلك مختبرات المنظمات غير الحكومية. وخلال التوسع الذي شهده المخطط، ازدادت معدلات الاستجابة المختبرية عن 90 % وتحسنت نسبة المختبرات التي أبلغت عن نتيجة زائغة واحدة على الأقل من 9.2 % (6/65) في عام 2005 إلى 5.4 % (17/316) في عام 2012.

**الدروس المستفادة:**

يتعين وجود دلائل إرشادية وطنية لإجراء الاختبارات ومختبر مرجعي بغية تنفيذ ضمان الجودة لخدمات اختبارات فيروس العوز المناعي البشري بشكل ناجح. وتعد التقييمات التي تنفذ في المواقع ذات أهمية حاسمة لدى جميع المختبرات المشاركة وهي المصدر الوحيد للرؤى بشأن أسباب النتائج الزائغة؛ والدروس التي يمكن للمختبر المرجعي تبادلها على الصعيد الوطني. ويساعد اختبار الكفاءة فنيي المختبرات على الاحتفاظ بمهارات اختبارات فيروس العوز المناعي البشري عن طريق ضمان تصديهم للعينات الإيجابية لفيروس العوز المناعي البشري بشكل منتظم.

## 摘要

**问题:**

在2005年之前，缅甸快速诊断性艾滋病病毒（HIV）检测质量都未得到监控，医院实验室也没有获得定期技术支持。

**方法:**

国家参考实验室引入了国家外部质量评估方案。方案涉及(i) 对实验室技术员进行艾滋病毒检测和质量评估系统要求的培训；(ii) 实施一年两次的熟练度专家组检测计划；(iii) 现场评估绩效不良的实验室以改善检测程序；(iv) 制定全国家导方针。

**当地状况:**

在2011年，缅甸总共有422家公共医院设有提供HIV检测的实验室。此外，非政府组织（NGO）支持的私人实验室也执行HIV检测。

**相关变化:**

2005年，该方案在65个公共实验室启动。在2012年，全国已经有347个实验室实施该方案，包括33个NGO实验室。在方案扩大期间，实验室响应率大于90%，实验室报告至少一例异常结果的比例从2005年的9.2%（6/65）降低至2012年的5.4%（17/316）。

**经验教训:**

成功实施艾滋病毒检测服务的质量保证需要国家检测指导方针和参考实验室。对所有参与实验室提供现场评估至关重要，这也是洞察异常结果原因的唯一措施；参考实验室的经验教训可以在全国分享。熟练度检测有助于实验室技术员通过确保经常接触艾滋病毒阳性样本来保持艾滋病毒检测的技能。

## Резюме

**Проблема:**

До 2005 года в Мьянме не осуществлялся контроль за качеством быстрой диагностики вируса иммунодефицита человека (ВИЧ), а лабораториям больниц не оказывалась регулярная техническая поддержка.

**Подход:**

Национальная справочная лаборатория внедрила национальную программу внешней оценки качества. Эта программа включала (i) обучение лаборантов тестированию на ВИЧ и требованиям системы оценки качества, (ii) реализацию полугодичной программы проверки квалификации, (iii) оценку на месте неудовлетворительно работающих лабораторий с целью совершенствования процедур тестирования и (iv) разработку национальных руководств.

**Местные условия:**

В 2011 году в общей сложности 422 государственные больницы в Мьянме располагали лабораториями, выполняющими тестирование на ВИЧ. Кроме того, тестирование на ВИЧ выполняли частные лаборатории, поддерживаемые неправительственными организациями (НПО).

**Осуществленные перемены:**

Реализация программы была начата в 65 государственных лабораториях в 2005 году. В 2012 году программа была распространена на всю страну и охватила 347 лабораторий, в том числе 33 лаборатории НПО. Во время расширения программы уровень участия лабораторий превышал 90%, а доля лабораторий, сообщивших по крайней мере об одном аберрантном результате, уменьшилась с 9,2% (6/65) в 2005 году до 5,4% (17/316) в 2012 году.

**Выводы:**

Для успешного обеспечения качества услуг тестирования на ВИЧ требуются национальные рекомендации и наличие справочной лаборатории. Проведение оценок на месте имеет решающее значение для всех участвующих лабораторий и является единственным источником для понимания причин аберрантных результатов. Этими выводами справочная лаборатория может поделиться на национальном уровне. Профессиональное тестирование поможет лаборантам поддерживать свои навыки тестирования на ВИЧ на должном уровне путем регулярного выявления ВИЧ-позитивных образцов.

## Introduction

Early diagnosis of human immunodeficiency virus (HIV) infection is needed to ensure timely access to care and prevent disease transmission.^1^ The emergence of rapid tests that detect HIV antibodies in body fluids has enabled the expansion of HIV diagnosis in resource-poor settings. However, many laboratory services remain inefficient because of a lack of equipment and technicians.^2^^,^^3^ There are concerns regarding testing accuracy, quality and interpretation of algorithms.^4^ Accurate HIV tests are essential for patient care and outcomes.^5^^,^^6^

Others have described some of the challenges of establishing national quality assessment schemes for HIV testing services.^7^^–^^11^ We report lessons learnt during eight years of establishing such a scheme in Myanmar.

## Approach

The Myanmar Ministry of Health provides laboratory services through the national reference laboratory which began to implement quality assessment in 2005 with technical and financial support from the Japan International Cooperation Agency. The scheme included four parts: (i) training workshops for laboratory personnel; (ii) an external proficiency panel testing programme for participating laboratories; (iii) on-site assessment by national reference laboratory staff; and (iv) development of national guidelines.

From 2005, the national reference laboratory conducted two cycles of training each year. The training was held at the reference laboratory and supported technically by the Japan International Cooperation Agency.

Biannually, the national reference laboratory sent five serum panels to all participating laboratories. The laboratories were expected to return test results within one month of receipt. The panels included strong-positive, weak-positive and negative HIV antibody samples. The number of positive and negative samples differed by panel. To maintain a satisfactory response rate, the national reference laboratory contacted every laboratory twice by phone, before sending the samples and afterwards to confirm receipt of samples. After assessing results from the participating laboratories, the national reference laboratory provided feedback by mail, including the proportion of the laboratories that kept the deadline.

In 2010, the Myanmar Ministry of Health legislated national guidelines for the scheme based on guidelines produced by the Joint United Nations Programme on HIV/AIDS.^12^ This was done to standardize the procedures and facilitate the expansion of the scheme.^13^ The guidelines describe each step in the scheme and the HIV testing procedures, including the serial testing algorithm for rapid tests. The guidelines propose that Alere Determine (Alere, Waltham, United States of America) be used for screening, Uni-Gold Recombigen® HIV-1/2 (Trinity Biotech, Wicklow, Ireland) for confirmation and HIV 1/2 STAT-PAK® (Chembio, Medord, USA) for second confirmation.^14^

## Local setting

There are 422 public hospitals in Myanmar, including six teaching hospitals, 28 general hospitals, 19 specialized hospitals, 45 district hospitals, and 324 township hospitals that provide HIV testing. In addition, private laboratories supported by nongovernmental organizations (NGOs) also provide HIV testing services. Between 2005 and 2012, the national reference laboratory selected 314 public hospital laboratories to be part of the scheme. Large hospitals were selected first. The number of HIV tests performed and availability of transportation and communication systems were also considered. Upon request, NGO-supporting laboratories were gradually included.

## Relevant changes

**Fig. 1:**
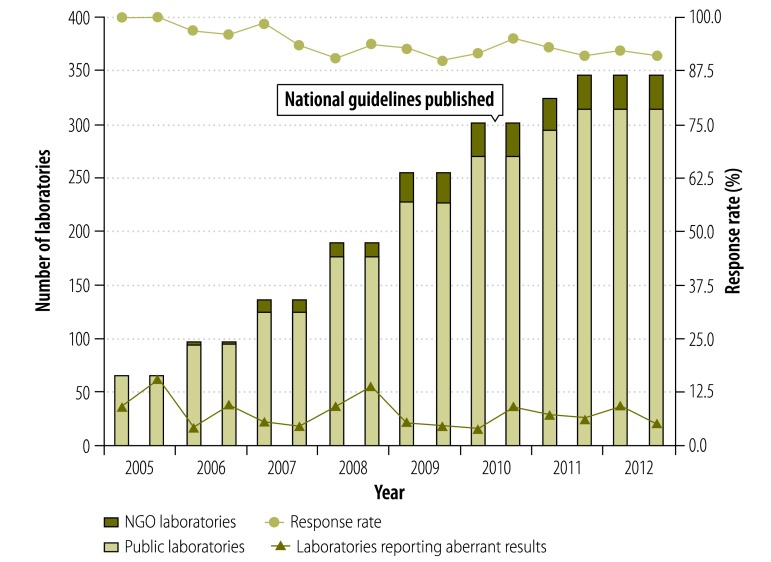
Data from the national external quality assessment scheme on human immunodeficiency virus testing, Myanmar, 2005–2012

The scheme was started in 65 laboratories in 2005 and gradually expanded to include 347 participating laboratories in 2012 (Fig. 1), which included almost all 330 townships in Myanmar. Of these laboratories, 33 were supported by NGOs. During the expansion of the scheme, laboratories’ response rates continued to be over 90% despite the inclusion of laboratories in remote areas with communication difficulties. The proportion of laboratories reporting at least one aberrant test result was improved from 9.2% (6/65) in 2005 to 5.4% (17/316) in 2012 (Fig. 1).

NGO: nongovernmental organization.

On-site assessment of poorly performing laboratories revealed some misunderstandings regarding HIV testing procedures, such as wrong incubation time or inadequate amount of sample being used (Box 1). We often observed that the test result was read immediately after the appearance of the control band, without allowing sufficient incubation time for a weak-positive sample to test positive. Most of the aberrant results (166/263) were false negatives. These results most frequently occurred in weak-positive samples that were diluted by the reference laboratory to cause a weak reaction. Thus, the weak-positive panel appeared to be effective for revealing misunderstandings relating to testing procedures. Some aberrant results were attributed to the lack of necessary equipment in laboratories, such as timers and micropipettes.

Box 1 **Reasons for aberrant rapid human immunodeficiency virus (HIV) diagnostic results, Myanmar, 2005–2012**

**Background factors causing false-negative results**

- Reading the test result before the instructed incubation time
- Use of an incorrect volume of the specimen and/or reagents
- Lack of experience in reading weak positive results

**Background factors causing false-positive results**

- Cross-contamination during the process of testing
- Reading the test result too long after the instructed incubation time
- Reading the test results obliquely from above

**Background factors that need to be addressed to obtain correct results**

- Misunderstanding the testing algorithm
- Clerical errors
- Poor quality of HIV test kit
- Insufficient equipment
- Not following the manufacturer’s instructions
- Using an expired test kit

The scheme found that nine types of test kit were used in the different laboratories and around 40% of the laboratories initially used a kit that had not been recommended in the guidelines. Most laboratories have now begun to use the recommended test kits, due to clearer guidance and improved procurement of HIV test kits resulting from increased funding.

## Discussion

A strong commitment by the national reference laboratory is crucial for any quality assurance programme.^4^ The Myanmar national reference laboratory has the mandate to promote and maintain the quality of all laboratory services and took full responsibility for implementing the scheme. However, we found that maintaining the scheme in a resource-poor setting required intensive efforts. For example, one of the most difficult tasks was to sustain a high response rate from the participating laboratories in remote areas. While expanding the scheme, smaller less-equipped laboratories located in remote areas needed technical assistance and arrangements to solve limitations in communication and postal infrastructure.

The national guidelines helped the national reference laboratory to implement the scheme by allocating responsibility and authority.^13^ The guidelines clearly describe the role of the national reference laboratory in the programme and require all public laboratories to participate in the scheme.

Maintaining the quality of HIV testing in the national reference laboratory itself was also important for the quality of the scheme. Therefore the laboratory was certified by an international external quality assessment scheme through the National Serology Reference Laboratory, Australia.^15^

### Proficiency testing

Proficiency testing programmes are an effective tool for improving the quality of laboratory services.^16^ Although they have some limitations, (such as incomplete assessment of the whole testing process and testing materials being treated differently to patient materials), these programmes remain useful. Compared with retests of samples from a participating laboratory performed by a reference laboratory, proficiency panel testing is a simpler and more feasible method to monitor quality in a resource-poor setting.

Acquiring and maintaining skills for HIV testing can be challenging for technicians in countries with a low HIV prevalence, such as Myanmar, because positive specimens are rarely encountered during everyday work. Proficiency testing helps technician to maintain skills by regularly identifying positive samples.

### On-site assessment

On-site assessments encourage problem solving and motivate staff. They are useful for improving the performance of health-care services.^17^ However, the assessments increase the workload of supervisors and supervisees, since they require time and resources. Thus, they were not suitable for all participating laboratories, but were an important component of the scheme. Supervisors from the national reference laboratory provided on-site training using proficiency samples. The findings from these on-site assessments generated practical information regarding the most probable causes of aberrant results. These findings were shared with all participating laboratories and were used to improve the content of training materials.

We also found that laboratories at local hospitals sometimes faced difficulties due to lack of commitment by the supervisors. We tried to solve this during on-site assessments. Sometimes we also provided simple equipment if it was the major cause of aberrant results.

### Running costs

The running costs and work burden associated with distributing proficiency sample panels often hinder the establishment of external quality assessment.^18^ In Myanmar, the sustainability of funding and the stretching of human resources at the national reference laboratory to maintain the scheme were a challenge. The annual cost of running the scheme in 350 laboratories was approximately 11 700 United States dollars (US$) in 2012, which included training (US$ 3000), panel preparation (US$ 4200), postage and communication (US$ 1000), report publications (US$ 500) and on-site assessment (US$ 3000).

### Conclusion

To ensure reliable HIV testing services in this context, assessment is needed. The on-site component is particularly important, as it is the only way to find the causes of aberrant results; information that can then be used to improve the performance of all laboratories in the country (Box 2).

Box 2 **Summary of main lessons learnt**

- Strong commitment by the national reference laboratory and supportive national guidelines are essential for the establishment of an external quality assessment scheme, especially in resource-poor settings.
- On-site assessment is crucial for all laboratories participating in the scheme, and the means by which the reference laboratory gains critical insight into the probable causes of aberrant results.
- Regular proficiency testing helps laboratories that rarely diagnose positive samples to keep their skills.

## References

[1] Marks G, Burris S, Peterman TA. Reducing sexual transmission of HIV from those who know they are infected: the need for personal and collective responsibility. AIDS. 1999;13(3):297–306. 10.1097/00002030-199902250-00001 10199219

[2] Birx D, de Souza M, Nkengasong JN. Laboratory challenges in the scaling up of HIV, TB, and malaria programs: The interaction of health and laboratory systems, clinical research, and service delivery. Am J Clin Pathol. 2009;131(6):849–51. 10.1309/AJCPGH89QDSWFONS 19461092

[3] Mashauri FM, Siza JE, Temu MM, Mngara JT, Kishamawe C, Changalucha JM. Assessment of quality assurance in HIV testing in health facilities in Lake Victoria zone, Tanzania. Tanzan Health Res Bull. 2007;9(2):110–4. 10.4314/thrb.v9i2.14312 17722413

[4] Parekh BS, Kalou MB, Alemnji G, Ou CY, Gershy-Damet GM, Nkengasong JN. Scaling up HIV rapid testing in developing countries: comprehensive approach for implementing quality assurance. Am J Clin Pathol. 2010;134(4):573–84. 10.1309/AJCPTDIMFR00IKYX 20855638

[5] Petticrew MP, Sowden AJ, Lister-Sharp D, Wright K. False-negative results in screening programmes: systematic review of impact and implications. Health Technol Assess. 2000;4(5):1–120. 10859208

[6] Shanks L, Klarkowski D, O’Brien DP. False positive HIV diagnoses in resource limited settings: operational lessons learned for HIV programmes. PLoS ONE. 2013;8(3):e59906. 10.1371/journal.pone.0059906 23527284 PMC3603939

[7] Chalermchan W, Pitak S, Sungkawasee S. Evaluation of Thailand national external quality assessment on HIV testing. Int J Health Care Qual Assur. 2007;20(2-3):130–40. 10.1108/09526860710731825 17585612

[8] Sushi KM, Gopal T, Jacob SM, Arumugam G, Durairaj A. External Quality Assurance Scheme in a National Reference Laboratory for HIV Testing in South India. World J AIDS. 2012;2(3):222–5 10.4236/wja.2012.23028

[9] Smit PW, Mabey D, van der Vlis T, Korporaal H, Mngara J, Changalucha J, et al. The implementation of an external quality assurance method for point- of-care tests for HIV and syphilis in Tanzania. BMC Infect Dis. 2013;13(1):530. 10.1186/1471-2334-13-530 24206624 PMC3830510

[10] Mashauri FM, Siza JE, Temu MM, Mngara JT, Kishamawe C, Changalucha JM. Assessment of quality assurance in HIV testing in health facilities in Lake Victoria zone, Tanzania. Tanzan Health Res Bull. 2007;9(2):110–4. 10.4314/thrb.v9i2.14312 17722413

[11] Chaillet P, Zachariah R, Harries K, Rusanganwa E, Harries AD. Dried blood spots are a useful tool for quality assurance of rapid HIV testing in Kigali, Rwanda. Trans R Soc Trop Med Hyg. 2009;103(6):634–7. 10.1016/j.trstmh.2009.01.023 19249069

[12] The Joint United Nations Programme on HIV/AIDS. Guidelines for organizing national external quality assessment schemes for HIV serological testing. UNAIDS/96.5. Geneva: World Health Organization; 1996. Available from: http://www.who.int/diagnostics_laboratory/quality/en/EQAS96.pdf [cited 2014 Oct 14].

[13] Guidelines on National External Quality Assessment for HIV Antibody Testing. Yangon: Myanmar Ministry of Health; 2010.

[14] WHO list of prequalified in vitro diagnostic products [Internet]. Geneva: World Health Organization; 2014. Available from: http://www.who.int/diagnostics_laboratory/evaluations/PQ_list/en/ [cited 2014 Jan 22].

[15] Gust A, Walker S, Chappel RJ, Dax EM. Anti-HIV quality assurance programs in Australia and the southeast Asian and Western Pacific regions. Accredit Qual Assur. 2001;6(4-5):168–72 10.1007/s007690000301

[16] Shahangian S. Proficiency testing in laboratory medicine: uses and limitations. Arch Pathol Lab Med. 1998;122(1):15–30. 9448012

[17] Bosch-Capblanch X, Garner P. Primary health care supervision in developing countries. Trop Med Int Health. 2008;13(3):369–83. 10.1111/j.1365-3156.2008.02012.x 18397400

[18] Alemnji G, Nkengasong JN, Parekh BS. HIV testing in developing countries: what is required? Indian J Med Res. 2011;134(6):779–86. 10.4103/0971-5916.92625 22310813 PMC3284089

